# Phase I Study to Assess the Safety, Tolerability and Pharmacokinetics of AZD4877 in Japanese Patients with Solid Tumors

**DOI:** 10.1111/j.1753-5174.2011.00034.x

**Published:** 2011-06

**Authors:** Taito Esaki, Takashi Seto, Hiroshi Ariyama, Shuji Arita, Chinatsu Fujimoto, Koichiro Tsukasa, Takuro Kometani, Kaname Nosaki, Fumihiko Hirai, Katsuro Yagawa

**Affiliations:** * Department of Gastrointestinal and Medical OncologyJapan; † Department of Thoracic Oncology, National Kyushu Cancer CenterFukuoka, Japan; § AstraZeneca KKOsaka, Japan

**Keywords:** AZD4877, Eg5 Inhibitor, Solid Tumors, Japanese

## Abstract

**Introduction:**

AZD4877 is a potent Eg5 inhibitor that has been shown to have an acceptable tolerability profile in a Phase I study of Western patients with solid tumors. This study was conducted to evaluate the safety, pharmacokinetic (PK) profile, maximum tolerated dose (MTD) and efficacy of AZD4877 in a Japanese population with solid tumors.

**Methods:**

In this Phase I, open-label, dose-escalation study, AZD4877 (10, 15, 20 or 25 mg) was administered as a 1-hour intravenous infusion on days 1, 8 and 15 of repeated 28-day cycles to Japanese patients with advanced solid tumors. Adverse events (AEs) were evaluated according to Common Terminology Criteria for Adverse Events (CTCAE) version 3.0. PK variables were assessed pre- and post dosing. The MTD of AZD4877 was determined by evaluating dose-limiting toxicities (DLTs). Efficacy was evaluated by assessing best response according to Response Evaluation Criteria In Solid Tumors version 1.0.

**Results:**

Of the 21 patients enrolled, 18 received at least one dose of AZD4877 (N = 3 in both the 10 and 15 mg cohorts, N = 6 in both the 20 and 25 mg cohorts). The most commonly reported AEs were fatigue and nausea (39% of patients each). One patient in each of the 20 and 25 mg cohorts experienced a DLT (neutropenia and febrile neutropenia). Dose escalation was halted at 25 mg and the MTD was not defined in this population. CTCAE grade ≥3 abnormal laboratory findings/vital signs were reported in 12 patients, with neutropenia (56%) and leukopenia (44%) being the most commonly reported. Exposure to AZD4877 was not fully dose proportional and AZD4877 clearance and elimination half-life appeared independent of dose. The best response to AZD4877 was stable disease in five of 16 evaluable patients.

**Conclusion:**

AZD4877 up to doses of 25 mg was well tolerated in Japanese patients. There was little evidence of clinical efficacy.

## Introduction

Kinesin spindle protein, also known as Eg5, is a microtubule motor protein essential for the formation of a bipolar mitotic spindle and normal centrosome separation during mitosis [1]. Eg5 expression is elevated in several proliferative tissues, including the thymus, tonsil, testis, esophageal epithelium and bone marrow, as well as in a number of solid tumors and leukemias [2–4]. Inhibition of Eg5 in replicating cells has been shown to prevent centrosomal separation and mitotic spindle assembly, which leads to the formation of monopolar spindles (“monoasters”), activation of the spindle checkpoint and mitotic arrest, resulting in cell death [1,5,6].

First generation antimitotic drugs, such as the taxanes and vinca alkaloids, have been shown to be effective anticancer agents [7]. However, by targeting tubulin, these agents affect the microtubular architecture of non-proliferating cells, which leads to a high incidence of neurotoxic side effects as well as myelosuppression [8,9]. Eg5 is not expressed in non-proliferating cells or in the adult peripheral nervous system, and so inhibition of Eg5 only targets actively mitotic cells [10]. Therefore, Eg5 inhibitors are not expected to cause the neurotoxic side effects commonly observed with traditional antimitotic agents [2,11]. Inhibition of Eg5 has been shown to cause cell death in a number of preclinical cancer cell lines and to have antiproliferative activity in human tumor xenograft models [11–15].

AZD4877 is a potent inhibitor of Eg5 that prevents centrosome separation and mitotic spindle assembly resulting in mitotic arrest, induction of apoptosis and cell death. AZD4877 was shown to inhibit growth of a broad panel of solid and hematologic tumor cell lines [16]. Furthermore, activity was observed in human tumor xenograft models including a primary bladder tumor model and the rituximab-insensitive non-Hodgkin's lymphoma model, DoHH2T53. In a Phase I study in Western patients, AZD4877 25 mg was shown to have an acceptable tolerability profile with evidence of monoaster formation suggesting proof of mechanism in a clinical setting. The best objective tumor response to AZD4877 was only stable disease [17].

The aim of this Phase I study was to evaluate the safety, pharmacokinetic (PK) profile, maximum tolerated dose (MTD) and efficacy of AZD4877 in Japanese patients with solid tumors.

## Methods

### Patients

Patients with advanced solid malignancies aged 20–75 years were included if they had a histologically or cytologically confirmed solid malignancy for which no standard curative or palliative measures existed or were no longer effective, and a World Health Organization (WHO) performance status of 0 or 1. Patients who had received prior treatment with an anticancer agent within 28 days of the first dose of study treatment (6 weeks for mitomycin treatment) were excluded from the study. Other exclusion criteria included absolute neutrophil count <1.5 × 10^9^/L, platelet count <100 × 10^9^/L, hemoglobin ≤9 g/dL, inadequate renal function (creatinine >1.3 × upper limit of normal [ULN]) or liver function (serum bilirubin >1.5 × ULN) as well as evidence of severe or uncontrolled systemic disease.

All patients provided written informed consent. The study was approved by the independent ethics committee for the trial center and was conducted in accordance with the Declaration of Helsinki [18].

### Study Design

**Figure 1:**
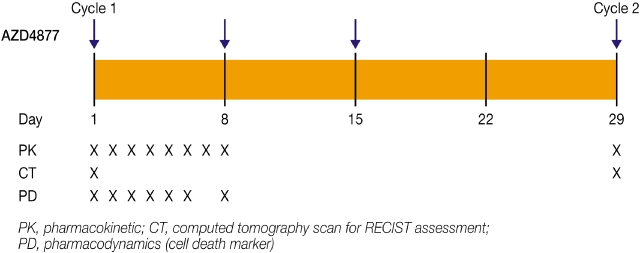
Study design.

This was a Phase I, open-label, single-center, dose-escalation study in Japanese patients (ClinicalTrials.gov identifier NCT00613652). AZD4877 was administered at escalating doses in separate cohorts of patients starting at 10 mg given as a 1-hour intravenous (iv) infusion on days 1, 8 and 15 of a 28-day cycle (Figure 1). The next patient cohort received the next highest dose level based on assessment of dose-limiting toxicities (DLTs). The MTD was identified as the highest dose at which no more than one of six patients experienced a DLT during cycle 1. Following completion of cycle 1, patients could receive consecutive treatment with AZD4877 if they continued to benefit and there was no evidence of disease progression.

### Objectives

The primary objectives of this study were to investigate the safety, tolerability and PK profile of AZD4877. The secondary objective was to evaluate the MTD of AZD4877 based on assessment of DLTs. Exploratory objectives included assessment of the antitumor activity of AZD4877 and evaluation of serum levels of the tumor cell death biomarkers M30 (caspase-cleaved cytokeratin 18) and M65 (cytokeratin 18).

### Assessments

Safety and tolerability were evaluated by assessment of adverse events (AEs) according to Common Terminology Criteria for Adverse Events (CTCAE) version 3.0. Laboratory assessments (clinical chemistry and hematology) were undertaken using samples collected pre-dose and on days 1, 8, 15 and 22 of cycles 1 and 2, and urinalysis was undertaken using samples collected pre-dose and on days 1, 8 and 15 of cycle 1 and on day 1 of subsequent cycles.

The MTD of AZD4877 was defined as the highest dose at which no more than one of six evaluable patients experienced a DLT during cycle 1. DLTs were defined as grade 3 or 4 hemolysis, grade 4 hematologic toxicity (thrombocytopenia or neutropenia >4 days), grade 3 or 4 neutropenia complicated by fever (oral temperature ≥38.5°C; axillary temperature ≥38.0°C), grade 2 diarrhea >4 days despite optimal management, grade 3 or 4 vomiting for >24 hours despite suitable anti-emetics or other grade 3 or 4 non-hematologic toxicity.

Blood samples for PK analysis were collected daily at various timepoints pre- and post-dose on days 1–8 of cycle 1, and day 1 of cycle 2. PK parameters evaluated included maximum plasma drug concentration (C_max_), plasma drug concentration 24 hours after administration (C_24h_), area under the concentration–time curve from zero to infinity (AUC_0–∞_), area under the concentration–time curve from zero to 24 hours (AUC_0–24h_), half-life associated with the terminal slope of a semi-logarithmic concentration–time curve (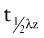), mean residence time (MRT), apparent volume of distribution at steady state (V_ss_) and total apparent plasma clearance (CL). Non-compartmental methods were used for the evaluation of the plasma concentration–time data and C_max_ was determined by inspection of the concentration–time profiles. Where possible, the terminal elimination rate constant (λ) was calculated by log–linear regression of the terminal portion of the concentration–time profiles, and 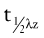 was defined as 0.6932/λ_Z_. AUC_0–24h_ was determined using the linear trapezoidal rule, and where appropriate, AUC_0–24h_ was extrapolated to infinity using λ to obtain AUC_0–∞_ values. MRT was calculated as the ratio of the area under the first moment curve (AUMC) to AUC plus the infusion duration/2. Total apparent clearance was calculated from the ratio of dose/AUC and V_ss_ was determined from the MRT × CL. The methods for the PK parameter assessments and calculations reported in this study have been described previously [19].

The tumor cell death biomarkers M30 and M65 have previously been shown to be elevated in patients with a variety of cancers and are considered to be measures of apoptosis and tumor burden. Blood samples for M30 and M65 biomarker assessment were taken pre-dose and at 24, 48, 72, 96 and 120 hours after infusion and on day 8 of cycle 1. Serum was extracted from whole blood samples (3 mL) by centrifugation (1,000 × *g* at 4°C) and stored at −20°C until required. Biomarker analysis was performed in duplicate by the Experimental Pharmacology Group, Paterson Institute of Cancer Research, University of Manchester (UK). Baseline corrected values were calculated by subtracting the mean of the two pre-dose values from the post-dose values.

Efficacy was determined by investigator assessment of overall best response according to Response Evaluation Criteria In Solid Tumors (RECIST) version 1.0. Baseline radiologic tumor assessments were performed within 28 days prior to administration of the first dose, and subsequent tumor evaluations were performed on day 29 of cycles 1 and 2 and subsequent odd numbered cycles. The minimum period of stable disease was defined as 8 weeks.

### Statistical Analysis

Data are summarized descriptively by dose group. Due to the limited number of patients expected in each dose group (N = 3–6 per group), no formal statistical comparisons between dose groups were planned or conducted.

## Results

### Patient Characteristics

A total of 21 patients were enrolled in this study during January 2008 to June 2009. Of the 21 patients, 18 received at least one dose of AZD4877 ranging from doses of 10 to 25 mg (Table 1). The mean age of patients was 62.7 years and 11 (61.1%) were female. All patients had advanced solid malignancies with a broad representation of primary tumor types, of which lung (33.3%) and breast cancer (16.7%) were the most common.

**Table 1:** Patient demographics and baseline characteristics

	AZD4877 dose†				
	
	10 mg (N = 3)	15 mg (N = 3)	20 mg (N = 6)	25 mg (N = 6)	Total (N = 18)
Age (years)					
Mean (SD)	66.7 (4.0)	62.7 (5.5)	62.2 (10.7)	61.3 (9.6)	62.7 (8.4)
Range	63–71	59–69	45–73	50–75	45–75
Gender, N (%)					
Male	2 (67)	2 (67)	1 (17)	2 (33)	7 (39)
Female	1 (33)	1 (33)	5 (83)	4 (67)	11 (61)
WHO performance status, N (%)					
Normal activity (0)	1 (33)	3 (100)	2 (33)	2 (33)	8 (44)
Restricted activity (1)	2 (67)	0	4 (67)	4 (67)	10 (56)
Primary tumor site, N (%)					
Lung	1 (33)	0	3 (50)	2 (33)	6 (33)
Breast	0	1 (33)	0	2 (33)	3 (17)
Pleura/pleural effusion	0	0	1 (17)	0	1 (6)
Prostate	0	1 (33)	0	0	1 (6)
Stomach	0	0	1 (17)	0	1 (6)
Colon	1 (33)	0	0	0	1 (6)
Other‡	1 (33)	1 (33)	1 (17)	2 (33)	5 (28)

† Dose assigned on day 1 of cycle 1.

‡ Other tumor sites include: thymus (2), ureter (1), renal/prostate (1) and primary unknown tumor (1).

SD = standard deviation; WHO = World Health Organization.

Seventeen of 18 patients discontinued study treatment due to lack of therapeutic response and one patient in the 20 mg dose cohort withdrew from the study due to an AE (grade 2 cystitis).

The median duration of treatment across all doses of AZD4877 was 50 days (range 15–163), with the longest median duration of 100 days (range 45–133) being in the 10 mg dose cohort. The median number of treatment cycles initiated across all doses was 2 (range 1–6). Of the 18 patients treated with AZD4877, only seven were continuing to receive their fully assigned dose by day 1 of cycle 2.

### Safety

All patients experienced at least one AE, of which the most commonly reported were fatigue (39%), nausea (39%), and pyrexia (33%) (Table 2). The majority of these were mild or moderate in intensity (CTCAE grade 1 or 2). Three patients experienced CTCAE grade ≥3 events; one in the 20 mg dose cohort experienced grade 4 neutropenia and two in the 25 mg dose cohort experienced grade 4 febrile neutropenia and grade 3 constipation, fatigue, urinary tract infection and anorexia, respectively (Table 3a). There were no deaths during the study.

**Table 2:** CTCAE of any grade reported in ≥20% of patients overall

	Patients with AEs, N (%)				
	AZD4877 dose				
	
	10 mg (N = 3)	15 mg (N = 3)	20 mg (N = 6)	25 mg (N = 6)	Total (N = 18)
Fatigue	1 (33)	1 (33)	2 (33)	3 (50)	7 (39)
Nausea	2 (67)	1 (33)	1 (17)	3 (50)	7 (39)
Pyrexia	0	3 (100)	2 (33)	1 (17)	6 (33)
Diarrhea	2 (67)	1 (33)	0	2 (33)	5 (28)
Dizziness	2 (67)	0	3 (50)	0	5 (28)
Rash	0	3 (100)	2 (33)	0	5 (28)
Constipation	1 (33)	0	1 (17)	2 (33)	4 (22)
Nasopharyngitis	0	0	2 (33)	2 (33)	4 (22)
Insomnia	1 (33)	0	2 (33)	1 (17)	4 (22)

**Table 3a:** CTCAE grade ≥3 events reported by any patient

	Patients with grade ≥3 event, N (%)				
	AZD4877 dose				
	
	10 mg (N = 3)	15 mg (N = 3)	20 mg (N = 6)	25 mg (N = 6)	Total (N = 18)
Any grade ≥3 event	0	0	1 (17)	2 (33)	3 (17)
Febrile neutropenia	0	0	0	1 (17)	1 (6)
Neutropenia	0	0	1 (17)	0	1 (6)
Constipation	0	0	0	1 (17)	1 (6)
Fatigue	0	0	0	1 (17)	1 (6)
Urinary tract infection	0	0	0	1 (17)	1 (6)
Anorexia	0	0	0	1 (17)	1 (6)

Two patients in the 20 and 25 mg cohorts experienced DLTs; CTCAE grade 4 neutropenia and febrile neutropenia, respectively. Grade 3 constipation, fatigue, urinary tract infection and anorexia were not considered to be DLTs because they occurred 1 week after the completion of cycle 1 and were not considered causally related to treatment by the investigator. The 20 and 25 mg dose cohorts were therefore expanded to six patients, however, no further DLTs were observed.

Twelve patients experienced CTCAE grade ≥3 abnormal laboratory findings/vital signs, the most common of which were neutropenia (56%) and leukopenia (44%). The incidence of neutropenia increased in an approximately dose-proportional manner, with five of six patients in the 25 mg dose cohort experiencing grade ≥3 neutropenia (Table 3b). Overall, the nadir in the laboratory hematology variables of white blood cell count, platelet count and absolute neutrophil count occurred by day 15 of cycle 1 (Table 3c). There were no other clinically relevant changes or trends in laboratory parameters or vital signs (WHO performance status, physical findings or ECG observations) over the dosing range.

**Table 3b:** CTCAE grade ≥3 abnormal laboratory findings/vital signs reported by any patient

	Patients with grade ≥3 abnormal laboratory findings/vital signs, N (%)				
	AZD4877 dose				
	
	10 mg (N = 3)	15 mg (N = 3)	20 mg (N = 6)	25 mg (N = 6)	Total (N = 18)
Any grade ≥3 finding/vital sign	1 (33)	1 (33)	4 (67)	6 (100)	12 (67)
Neutropenia	1 (33)	1 (33)	3 (50)	5 (83)	10 (56)
Leukopenia	0	0	3 (50)	5 (83)	8 (44)
Lymphopenia	0	0	0	1 (17)	1 (6)
Thrombocytopenia	0	0	0	1 (17)	1 (6)
Blood sodium decreased	0	0	1 (17)	0	1 (6)
Hemoglobin decreased	0	0	0	1 (17)	1 (6)
Lymphocyte count decreased	0	0	0	1 (17)	1 (6)
Weight decreased	0	0	1 (17)	0	1 (6)

Cut-off value for values for defining grade ≥3 abnormal laboratory finding: neutropenia <1.0 × 10^9^/L; leukopenia <2.0 × 10^9^/L; lymphopenia: <0.5 × 10^9^/L; thrombocytopenia: <50.0 × 10^9^/L; blood sodium decreased: <130 mmol/L; hemoglobin decreased: <8.0 g/dL; lymphocyte count decreased: <0.5 × 10^9^/L; weight decreased: ≥20% from baseline.

**Table 3c:** Selected laboratory hematology variables at baseline and during cycle 1

	AZD4877 dose				
	
Parameter, mean (SD)	10 mg (N = 3)	15 mg (N = 3)	20 mg (N = 6)	25 mg (N = 6)	Total (N = 18)
White blood cell count, 10^3^/µL					
Baseline	4.28 (0.91)	6.95 (1.46)	6.03 (3.08)	4.94 (1.28)	5.53 (2.11)
Cycle 1, Day 8	4.13 (0.63)	5.50 (1.48)	2.97 (1.06)	3.01 (0.99)	3.60 (1.37)
Cycle 1, Day 15	3.59 (0.69)	4.66 (2.19)	2.82 (1.20)	1.58 (0.57)	2.84 (1.55)
Cycle 1, Day 22	4.09 (0.66)	4.53 (1.70)	2.64 (0.69)	2.73 (0.68)	3.23 (1.14)
Platelet count, 10^3^/µL					
Baseline	233.3 (69.3)	242.3 (21.5)	279.2 (40.2)	276.5 (71.7)	264.5 (54.7)
Cycle 1, Day 8	227.3 (36.7)	229.0 (35.9)	245.7 (45.5)	246.5 (99.7)	240.1 (62.6)
Cycle 1, Day 15	203.3 (46.5)	217.0 (6.1)	265.2 (79.5)	184.5 (85.1)	219.9 (73.9)
Cycle 1, Day 22	205.0 (54.0)	232.0 (7.9)	262.2 (48.0)	308.7 (117.3)	263.1 (81.1)
Total ANC, 10^3^/µL					
Baseline	2.48 (0.38)	4.36 (0.87)	4.34 (2.95)	3.39 (1.38)	3.72 (1.94)
Cycle 1, Day 8	2.00 (0.37)	3.23 (1.26)	1.81 (0.99)	2.00 (0.97)	2.14 (1.01)
Cycle 1, Day 15	1.25 (0.27)	2.33 (1.62)	1.49 (0.82)	0.57 (0.50)	1.28 (0.99)
Cycle 1, Day 22	1.68 (0.35)	2.30 (1.50)	1.25 (0.69)	1.41 (0.48)	1.55 (0.79)

ANC=absolute neutrophil count.

### Pharmacokinetics

**Figure 2:**
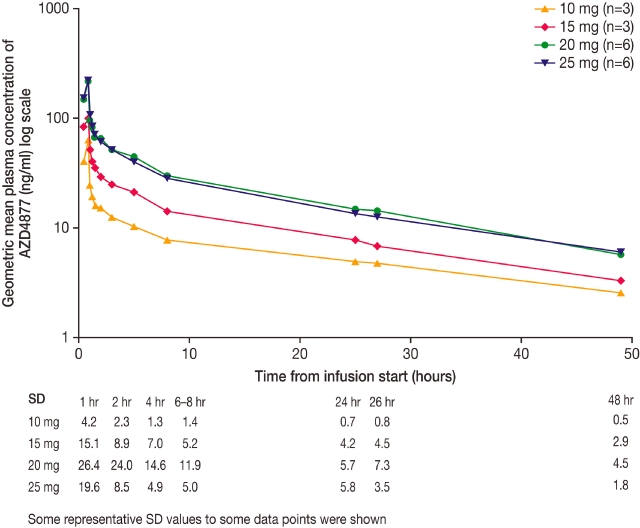
Geometric mean plasma concentration of AZD4877 versus time by dose level following single dosing. SD=standard deviation.

Systemic exposure to AZD4877 (as calculated by C_max_, AUC_0–∞_, C_24h_ and AUC_0–24h_) increased in an approximately dose-proportional manner in the 10 and 15 mg cohorts, however, greater than dose-proportional increases were observed between the 10 and 20 mg cohorts and little change was observed between the 20 and 25 mg cohorts (Figure 2). MRT, 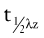 and V_SS_ values remained relatively unchanged in the 15, 20 and 25 mg cohorts, however, higher values for each of these parameters were reported in the 10 mg cohort (Table 4). Clearance of AZD4877 was similar in the 10, 15 and 25 mg cohorts (19.5 to 22.4 L/h) but lower in the 20 mg cohort (14.0 L/h).

**Table 4:** Pharmacokinetic parameters of AZD4877 during cycle 1

	AZD4877 dose			
	
PK parameter	10 mg (N = 3)	15 mg (N = 3)	20 mg (N = 6)	25 mg (N = 6)
C_max_ (ng/mL)	63.4 (22.4)	99.7 (59.0)	218.0 (51.2)	224.0 (15.8)
C_24h_ (ng/mL)	4.9 (13.4)	7.8 (55.3)	14.9 (38.9)	13.6 (47.8)
AUC_0–∞_ (ng·h/mL)	447.0 (20.0)	671.0 (60.5)	1430.0 (33.5)	1280.0 (19.4)
AUC_0–24h_ (ng·h/mL)	234.0 (17.6)	424.0 (45.3)	882.0 (32.3)	838.0 (15.9)
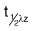 (h)	28.7 (9.0)	20.3 (32.5)	21.3 (45.9)	21.1 (27.7)
MRT (h)	34.2 (8.9)	22.9 (36.3)	22.4 (59.7)	22.2 (26.1)
V_SS_ (L)	766.0 (11.6)	512.0 (37.3)	314.0 (52.3)	432.0 (24.6)
CL (L/h)	22.4 (20.0)	22.3 (60.5)	14.0 (33.5)	19.5 (19.4)

Data are expressed as geometric mean (CV%).

AUC_0–∞_ = area under the plasma concentration–time curve from zero to infinity; AUC_0–24h_ = area under the plasma concentration–time curve from zero to 24 hours; C_24h_ = plasma drug concentration 24 hours after administration; CL = total body clearance of drug from plasma; C_max_ = maximum plasma drug concentration; CV = coefficient of variation; MRT = mean residence time; SD = standard deviation; 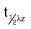 = half-life associated with the terminal slope (λz) of a semi-logarithmic concentration–time curve; V_SS_ = volume of distribution at steady state.

### Biomarkers

**Figure 3:**
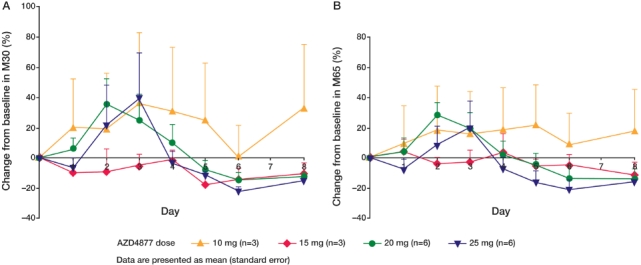
Mean change from baseline (%) in (A) M30 and (B) M65 values within the first 8 days of cycle 1.

**Figure 4:**
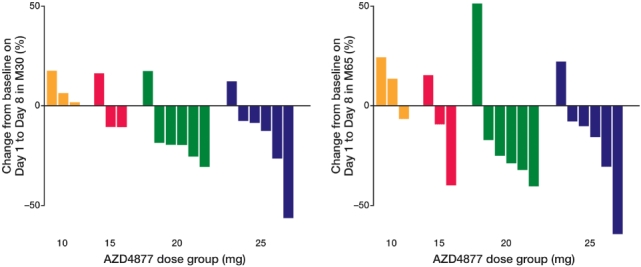
Change (%) in (A) M30 and (B) M65 from baseline on day 1 to day 8 for individual patients.

Overall, there was an approximately 15% mean decrease from baseline in the tumor cell death biomarkers, M30 and M65 in the 15, 20 and 25 mg dose cohorts over the 8-day assessment period of cycle 1. Consistent with this finding, peaks in these biomarkers were observed in the 20 and 25 mg dose cohorts on days 1 and 2, potentially indicating waves of drug-induced cell death at the earlier timepoints. A different profile was observed for the 10 mg dose with no reduction in levels of M30 or M65 by day 8. Mean percentage changes from baseline for M30 and M65 values within the first 8 days of therapy are shown in Figure 3. In the AZD4877 20 and 25 mg cohorts, decreases in both biomarkers were observed from days 3 to 8. Furthermore, changes from baseline of >30% were observed for several patients over the 8-day assessment period (Figure 4).

### Efficacy

No patients experienced a complete or partial response. The overall best response was stable disease ≥8 weeks, which was observed in five of 16 evaluable patients; two in the 10 mg cohort (one lung and one ureter cancer), one in the 15 mg cohort (renal/prostate cancer) and two in the 20 mg cohort (one lung and one thymus). The patient in the 20 mg cohort with thymus cancer had stable disease for ≥12 weeks.

## Discussion

AZD4877 monotherapy at doses up to 25 mg weekly was generally well tolerated in Japanese patients with a range of solid tumors. The most commonly reported CTCAE grade ≥3 abnormal laboratory findings/vital signs was neutropenia, which was dose related. Two patients in the 20 and 25 mg cohorts experienced DLTs, of grade 4 neutropenia and febrile neutropenia, however, there were no more DLTs when the cohorts were expanded. In a parallel US study, patients with solid tumors receiving AZD4877 30 mg experienced neutropenia, which led to 25 mg being defined as the recommended dose for further evaluation [17]. Consequently, the MTD for AZD4877 was not defined in Japanese patients.

Systemic exposure to AZD4877 was not fully dose proportional, which may be due to the small number of patients in each cohort. Despite this, the PK parameters for AZD4877 in Japanese patients were comparable with those reported in Western patients [17]. Although reductions in the levels of the tumor cell death biomarkers M30 and M65, possibly due to a small reduction in tumor burden, were noted within the first 8 days of therapy the best response to AZD4877 was stable disease ≥8 weeks. Similarly, the best response to weekly treatment with AZD4877 in Western patients was also stable disease ≥12 weeks [17]. This limited clinical response is similar to that reported for other Eg5 inhibitors [20–25], and may be due to an insufficient therapeutic window or drug-resistant Eg5 mutations.

In conclusion, treatment with AZD4877 is feasible in Japanese patients with advanced solid tumors. The best objective tumor response to AZD4877 was only stable disease. Because of the relative lack of clinical efficacy in this and other completed Phase I and II trials of AZD4877, further development of this agent is not planned.
